# Effect of Strength Training on Body Composition, Volumetrics and Strength in Female Breast Cancer Survivors

**DOI:** 10.3390/healthcare13010029

**Published:** 2024-12-27

**Authors:** Rocío Pardo-Hernández, Jessica Fernández-Solana, Jerónimo J. González-Bernal, Ena Monserrat Romero-Pérez, Mario Alberto Horta-Gim, Luis Enrique Riojas Pesqueira, María Nieves Muñoz-Alcaraz, Josefa González-Santos, Mirian Santamaría-Peláez

**Affiliations:** 1 Department of Health Sciences, University of Burgos, 09001 Burgos, Spain; rph1001@alu.ubu.es (R.P.-H.); jejavier@ubu.es (J.J.G.-B.); mjgonzalez@ubu.es (J.G.-S.); mspelaez@ubu.es (M.S.-P.); 2 Division of Biological Sciences and Health, University of Sonora, Hermosillo 83000, Mexico; ena.romero@unison.mx (E.M.R.-P.); mario.horta@unison.mx (M.A.H.-G.); enrique.riojas@unison.mx (L.E.R.P.); 3 Córdoba and Guadalquivir Health District, Andalusia Health Service, 14011 Córdoba, Spain; marian.munoz.sspa@juntadeandalucia.es; 4 Maimónides Biomedical Research Institute of Córdoba (IMIBIC), Reina Sofía University Hospital, University of Córdoba, 14004 Córdoba, Spain

**Keywords:** breast cancer, radical mastectomy, lymphoedema, body composition, muscle strength, survivorship

## Abstract

Background/Aims: This cross-sectional study investigates body composition and strength in female breast cancer survivors, focusing on the effects of radical mastectomy and the presence of upper extremity lymphoedema. The main objective was to understand body composition, volumetry, and strength, as well as response to strength training in female breast cancer survivors. Methods: Twenty-three women (aged 42–74 years old) with radical mastectomy in the last five years were assessed by measuring body composition (weight, water percentage, fat, muscle, and lean mass), maximal strength, perimeters, and brachial volumes. Participants completed a 10-week strength training program of moderate intensity with 20 training sessions. No significant differences were found between the affected/healthy hemispheres in terms of composition, perimeters, and volumetrics. However, 11 women were found to have lymphoedema (47.8%). No statistically significant differences were found between hemibodies after the intervention, although improvements were obtained in pectoral strength and manual grip, as well as in muscle mass and lean mass [*p* = 0.002 each]. Cases with lymphoedema were reduced to 5 (21.73%). Conclusions: While strength training is shown to benefit body composition, strength, and the incidence of lymphoedema in mastectomized women, further scientific evidence is needed with larger controlled trials and follow-up studies to validate these findings, as well as the impact on the quality of life of these survivors.

## 1. Introduction

Breast cancer is one of the most common malignancies and a leading cause of morbidity and mortality worldwide. According to the World Health Organization (WHO), 9.6 million cancer-related deaths were recorded in 2018, underscoring the substantial global burden of this disease and the need for effective preventive and therapeutic strategies. In Spain, the National Institute of Statistics (INE) identifies cancer as the second leading cause of death, accounting for approximately 26.4% of all deaths [1]. Within the Spanish context, data from the Global Cancer Observatory (GCO) indicate that in 2021, 285,530 new cases of cancer were diagnosed, including 34,333 cases of breast cancer, the most common malignancy among women [2,3]. It is estimated that one in eight women in Spain will develop breast cancer, particularly between the ages of 46 and 65, and this tumor type is responsible for about 5.8% of annual cancer deaths in women. Although the five-year relative survival rate approaches 89.2%, about 30% of women diagnosed at early stages will suffer relapse with distant metastasis [4,5,6]. These statistics highlight the complex clinical landscape faced by survivors, who must contend not only with the threat of recurrence but also with ongoing treatment-related complications

Although it has increased in recent years, it accounts for only 1% of all breast cancer cases and is responsible for 0.1% of cancer deaths in men [7,8]. In Europe, the incidence of male breast cancer is 1 case per 100,000 population, affecting mostly men in their 70s and 80s, with a peak incidence at age 71 [9,10,11].

Among these complications, lymphedema stands out as one of the most frequent and debilitating sequelae, primarily affecting the upper limb on the side of surgical intervention, such as mastectomy. Lymphoedema can cause swelling, pain, and decreased arm functionality, directly impacting patients’ daily activities, autonomy, and overall quality of life [12,13,14]. This condition, estimated to affect about 20–30% of women treated for breast cancer, is chronic and difficult to reverse, underscoring the urgency of effective prevention and management strategies [15,16]. In this context, there is growing interest in identifying modifiable risk factors and rehabilitation approaches that can mitigate the severity and progression of lymphedema.

Body composition has emerged as a potentially critical factor in this regard. Recent research suggests that body mass index (BMI), fat mass, and muscle mass may influence both the predisposition to develop lymphoedema and its overall severity [17,18]. A higher percentage of fat mass has been associated with increased complications, whereas adequate muscle mass may act as a protective factor. Understanding this relationship is essential to identify high-risk patients and guide more precise interventions. However, current evidence remains limited and predominantly focused on BMI without thoroughly examining lean and muscle mass as potentially decisive elements in the onset and progression of lymphedema [19,20]. This gap in the literature hinders the development of targeted interventions designed to optimize body composition and reduce lymphedema risk.

Systematic measurement of muscle strength and body composition in breast cancer survivors can provide valuable insights into their functional status, informing more personalized rehabilitation programs. These measurements not only help monitor patient progress and the effectiveness of interventions but also facilitate adjustments to exercise regimens [21,22]. In addition, it has been shown that strength training, when introduced in a controlled and progressive manner, can offer multifaceted benefits: improving lymphatic circulation, strengthening muscle mass, and potentially reducing fluid accumulation, all of which may lessen lymphedema-related symptoms and promote a more complete functional recovery [23,24,25]. Such exercise-based interventions also hold promise for enhancing cardiorespiratory capacity, alleviating fatigue and depressive symptoms, and ultimately improving survivors’ quality of life.

A recent systematic review has clarified strength-training parameters suitable for breast cancer survivors: moderate-to-high intensity (60–80% of one-repetition maximum), 2 to 3 sets of each exercise, and sessions of about 20–60 min. Implementing these guidelines can improve muscular strength, quality of life, and mental well-being while reducing fatigue and depression. Importantly, these training protocols may also help in controlling lymphedema. All this improves strength and quality of life and reduces fatigue and depression levels. It also improves cardiorespiratory capacity and reduces lymphedema. However, the review did not report in detail how strength training affects body composition, leaving a critical area of investigation unexplored [26].

Therefore, this study aims to analyze body composition, limb volumetry, and muscular strength in female breast cancer survivors, as well as their response to a ten-week strength training program. Additionally, it seeks to determine whether such training can enhance physical condition and reduce the incidence or severity of lymphedema, thereby providing a stronger scientific basis for individualized exercise interventions in clinical practice. The hypotheses proposed are that this training program will improve participants’ body composition, increase their strength, and yield volumetric benefits that reduce the incidence and impact of lymphedema.

## 2. Materials and Methods

### 2.1. Participants

To understand the physical characteristics of breast cancer survivors and their response to strength training, we conducted a longitudinal study with a total of 23 women who underwent radical mastectomy. This surgical procedure was first described in the late 19th century by Halsted, whose current protocol was established in 1972 by Madden [27], according to which the surgery involves ablation of the entire breast as well as the axillary lymphatic tissue [28].

The study design was longitudinal, consisting of pre-test assessment, intervention, and post-test assessment. No control group was recruited; however, comparisons were made between affected and healthy hemi bodies. The sampling was non-probabilistic and non-random methods, being selected for convenience through an open invitation to breast cancer survivors’ associations in Sonora, Mexico, who had to meet eligibility criteria.

Inclusion criteria were having been diagnosed with breast cancer, having undergone radical mastectomy, having less than five years since their last surgery, and having voluntarily completed the informed consent form. Exclusion criteria included failure to perform mastectomy, current upper extremity infection, presence of lymphangitis, and failure to comply with informed consent.

### 2.2. Procedure

The variables assessed were height, weight, percentage of fat mass, fat mass in kilograms, muscle mass, lean mass, percentage of water, manual grip strength of the affected and healthy sides, maximum strength in chest press, maximum strength of bilateral pectoral contraction, maximum strength of unilateral pectoral contraction of the healthy and affected sides, as well as perimeters and volumetrics of both arms.

Body composition analysis included measurements of weight, height, percentage of fat mass, kilograms of fat mass, lean mass, muscle mass, and percentage of water. This analysis was carried out using a professional scale model, TANITA SC331S, used in different populations [29,30], scrupulously following the user manual. Grip strength was measured using a dynamometer, taking three measurements and determining the arithmetic mean.

The evaluation of the maximum force (1RM) of unilateral, bilateral chest contraction and chest press was carried out using the protocol described by Tanori et al. [31] on a multi-station machine (EXM2500Sv Body-Solid). For the determination of the maximum force in unilateral pectoral contraction, we started with an initial load equivalent to 10% of the body weight of each participant, making increments of 5% until the maximum load that each participant was able to mobilize on a single occasion was individually established. For the determination of maximum strength in chest press and bilateral pectoral contraction, we started with an initial load of 20% of body weight. After each successful attempt, a 10% increase in load was applied.

These increments were applied as a function of perceived exertion using the OMNI-RES scale [31], with two minutes of recovery between each attempt. If load shifting was achieved twice, the load increase was continued; if not, the weight was decreased by 5% to achieve greater accuracy.

On the other hand, the assessment of arm circumferences was performed using the procedure employed by Tánori et al., 2020 [32]. Accordingly, measurements were performed while seated, with the shoulder in flexion and abduction of approximately 30°, extending the arm in a relaxed manner on a table, with the elbow in extension, and the forearm in a supinated posture. Depending on the length of each participant’s arms, 10 to 12 points were marked on the skin, spaced every 4 cm starting from the distal wrist crease to the region near the axilla. Circumference measurements were taken perpendicular to the main axis of the limb, with a retractable tape and without pressing on the skin.

Taking the perimeter measurements of the participants’ limbs as a reference, the volumetrics of the segments of each arm were found, allowing the calculation of the total volume of the limbs. To do this, starting from each of the perimeters, the brachial radius of each measurement point was calculated using the equation:

$$r\;=\;\frac{P}{2\pi }$$

The height of each segment was then determined by taking the four centimeters between measuring points as the generatrix. Finally, using the calculated data, the volume of each segment of the arms was found using the mathematical formula for the truncated cone [33].

$$h\;=\;\sqrt{g^{2}\;+\;{\left(r2\;-\;r1\right)}^{2}}$$

$$Volume\;=\;\frac{h\pi }{3}\;(r1\;+\;r2\;+\;r1r2)$$

Once the volumetric data for all upper limb segments were available, the output variables were calculated by summation: forearm volume, upper arm volume, and total upper limb volume. The measurement of perimeters and subsequent calculation of volumetrics has proven to be a highly appropriate and reliable practice [34,35].

The diagnostic criteria for lymphoedema were followed, indicating the presence of lymphoedema if there is a volumetric difference between the two upper limbs of at least 200 milliliters [36].

### 2.3. Intervention

The strength training program began the week after the initial evaluations. The intervention was carried out over 10 weeks, with two weekly sessions separated by at least 48 h. All training days had the following structure: initially, an aerobic warm-up was carried out, followed by strength training, with a 5-min break after the warm-up and between each of the exercises.

The warm-up consisted of 30 min of aerobic exercise on a static bike (except in the first two weeks, in which 20 and 25 min were performed, respectively, to promote adaptation). Resistance was chosen by the participants since it is not the objective of the study.

After the 5 min of rest, strength training was carried out. For this, the same machines with which they were evaluated were used. The exercises, separated by 5-min breaks, were performed in the following order: pectoral press, bilateral chest contractor, and unilateral pectoral contractor. As in the evaluation, the exercises were executed as follows:

The pectoral press was executed in a seated position, with the back and head on the back of the machine, the shoulders in the abduction of approximately 80°, and elbows in flexion of 90°. The load is propelled in a ventral direction up to the full extension of the elbows.

The bilateral contraction begins in a sitting position, with the back and head resting on the machine, shoulders in 90° abduction, and elbows in 90° flexion. The forearm in pronation is placed on the padding of the machine, and the movement is executed in frontal adduction until the arms are joined in the sagittal plane.

Three sets of each exercise were performed and separated by one minute of recovery. The sets consisted of 12-10-12 repetitions during one week and 16-13-16 repetitions the following week maintaining the load. Every two weeks, the load was progressively increased. The initial load was 50% of the 1RM in the chest press exercise and 40% in the chest contractor. The progression of loads to every two weeks was 5% of 1RM, concluding the program with a load of 75% and 65% of 1RM, respectively, which implies a moderate intensity. This progression of loads was determined following the recommendations of the American College of Sports Medicine (ACSM) [36], which have been used in studies with this population.

### 2.4. Statistical Analysis

All statistical analyses were performed using SPSS version 25 (IBM Inc., Chicago, IL, USA). Firstly, socio-demographic and body composition characteristics were analyzed using descriptive analyses. Qualitative variables were shown by frequencies and percentages; quantitative variables were studied through means and Standard Deviations (*SD*). Before proceeding to inferential analyses, a Shapiro–Wilk normality analysis was performed. Those variables with a normal distribution were tested using parametric tests (Student’s *t*-test for related and independent samples), while variables with a non-normal distribution were compared using non-parametric analyses (Mann Whitney U and Wilcoxon signed-rank test). All the analyses carried out took 95% as a confidence index, with a statistical power of 0.05.

This study is subject to current biomedical research legislation and the principles of the Declaration of Helsinki. Written informed consent was collected from all participants prior to the start of the study. Approval was obtained from the ethics committee of the University of Sonora (DMCS/CBIDMCS/D-50).

## 3. Results

The sample comprising the present study consisted only of women (*n* = 23), with a mean age of 55.70 years and a mean BMI of 27.72 (Table 1). Most of the participants (56.5%) were diagnosed and mastectomized on the right side of the body, with one participant undergoing a double-radical mastectomy. The presence of lymphoedema was identified in 11 participants, representing 47.8% of the sample (Table 2).

The differences in perimeter measurements of both upper limbs were not statistically significant in any of the cases, as can be seen in Table 3 and Table 4.

With regard to upper limb volumes, no significant differences were found in this sample, neither in the forearm [t(44) = 0.511, *p* = 0.612], in the upper arm, nor in the whole upper limb. The comparison of unilateral pectoral strength and manual grip also showed no significant differences (Table 5).

Similarly, after the intervention, no significant differences were found in the forearm [t(44) = 0.035, *p* = 0.972], arm, or full upper limb volumes, as well as in unilateral pectoralis or manual grip strength.

In addition, intergroup comparisons were established to determine the existence of differences in the characteristics of body composition and maximum strength in chest press and bilateral chest contraction with respect to the presence or absence of lymphoedema (Table 6 and Table 7). In this case, no significant differences were found in any of the variables, so it could be concluded that the presence of lymphoedema does not imply differences in body composition or bilateral maximum strength measurements.

The number of people affected by lymphoedema was calculated, with a total of 5 people maintaining lymphoedema after the intervention (21.73%). Intra-group comparisons showed significant differences in unilateral pectoral strength of affected and healthy limbs, as well as in the grip of both arms. Differences in volume were not significant in the forearm, upper arm, or whole upper limb. Differences in muscle mass and lean mass were significant, as were differences in fat mass and body water.

Finally, the differential scores of the variables with significant responses to treatment were analyzed with respect to the affected or healthy hemibody. No significant differences were found in unilateral pectoral strength [U = 0.440, *p* = 0.660], grip strength [U = −0.158, *p* = 0.875], muscle mass [t(44) = 0.205, *p* = 0.838], or lean mass [t(44) = 0.206, *p* = 0.838] (Table 8 and Table 9).

## 4. Discussion

The present cross-sectional study aimed to analyze the body composition, volume, and strength of 23 female breast cancer survivors, as well as their response to a ten-week strength training program. In our results, the comparison of perimeters showed no significant differences between hemispheres. The same was true for the forearm, upper arm, and whole upper limb volume.

Likewise, following the diagnostic criteria, the analyses performed showed the presence of lymphoedema in 47.8% of the sample; however, this condition did not imply differences in terms of body composition or maximum strength in chest press or bilateral pectoral contraction.

The mean BMI of the participants was 27.72 kg/m^2^, with 12 of them being overweight. This is consistent with the findings of San Felipe et al. [37], who noted the high frequency of overweight and obesity in women breast cancer survivors, especially those undergoing chemotherapy. This factor, they note, worsens the prognosis of breast cancer, increasing the mortality rate. Albuquerque et al. [38] have also confirmed the high rate of obesity and overweight, as well as the correlation between this variable and the increase in pain experienced.

In contrast, other authors, such as Schlesinger et al. [39], showed the obesity paradox, according to which a certain degree of overweight acts as a protector against mortality, although without going into the origin of the overweight, which could be due in these cases to a high muscle mass, with the lack of skeletal muscle being the cause of the complications. This is the assumption demonstrated by Caan et al. [40] in their trial. In our study, the mean muscle mass showed slight differences between people with lymphoedema and those without lymphoedema, although these differences were not significant.

Similarly, McClain et al. [41] corroborated how comprehensive analysis of body composition is critical in breast cancer, having found associations that fat mass and lean mass are associated to a greater degree than BMI with the metabolites under study. However, in our study, lean mass and fat mass did not differ significantly between those with lymphoedema and those without.

After intervention, differences were found in muscle mass and lean mass, as opposed to fat mass and water percentage. Improvements were also found in the strength of both pectoral muscles, as well as in the grip of both hemibodies. Improvements were modest, possibly due to the brevity of the intervention, and were being performed with whole-body composition measurements, which may not be affected by partial training limited to specific muscle groups. However, those affected by lymphoedema were reduced from 11 (47.8%) to 5 (21.73), reflecting the remarkable effect of this training.

This is consistent with the findings of Fonnegra et al. [42], although aerobic endurance was not assessed in our study, according to which strength and endurance intervention for at least six months generate positive effects on strength and body composition in breast cancer survivors. These interventions also lead to improvements in cardiorespiratory fitness and other physiological variables, which in turn lead to improved physical and psychological fitness in these individuals.

This study was limited by the small sample size, and further expansion of the trial is recommended. In addition, intensity and endurance were not assessed using rigorous criteria. Although comparisons were made between both hemibodies, it would be advisable to expand this field of research in the future with control groups since body composition variables could not be controlled in the present study. Furthermore, it would be advisable to expand the available evidence on the influence of body composition on the development of lymphoedema and other specific complications since the literature consulted is limited, on most occasions, to participants’ weight and BMI, but not much evidence has been found regarding muscle, lean and fat mass, as well as other variables of interest, such as bone density. Consequently, future research should consider the study of strength interventions in breast cancer survivors with large, controlled groups and include variables such as bone density, cardiorespiratory capacity, and psychological and functional variables since they determine, in turn, another of the fundamental variables to be taken into account: the quality of life of these people.

## 5. Conclusions

Individuals with lymphoedema accounted for half of the sample, corroborating the high incidence of this complication. Intergroup analyses showed no conclusive differences between people with and without lymphoedema in terms of weight, BMI, lean, muscle and fat mass, and maximal pectoral strength in different measurements. However, after strength training, improvements in lean and muscle mass, as well as in pectoral and grip strength of both hemispheres, were observed.

However, it is necessary to go deeper into this field, developing research with a larger sample size and control group to ensure whether or not there are associations that can help us identify and treat these cases in order to provide survivors with a better quality of life.

## Figures and Tables

**Table 1:** Description of the body characteristics of the sample.

	*M*	*SD*
Age	55.70	9.508
Height	159.78	6.557
BMI	27.72	3.64
Weight	71.14	12.17
Fat mass (percentage)	37.23	8.01
Fat mass (kg)	27.29	9.04
Muscle mass	41.617	3.93
Lean mass	43.85	4.12
Water percentage	43.81	4.90

Mean (*M*), Standard Deviation (*SD*).

**Table 2:** Clinical data of the sample.

	*N*	*%*
Operated side		
Right	13	56.5
Left	9	39.1
Both	1	4.3
IMC		
Normal weight	5	21.7
Overweight	12	52.2
Obesity	6	26.1
Lymphedema		
Yes	11	47.8
No	11	47.8

**Table 3:** T-test independent samples. Perimeter measurement differences.

	Affected Hemibody		Healthy Hemibody		t-Student Independent Samples		
	*M*	*SD*	*M*	*SD*	*T*	*p*	Effect Size
Perimeter 1	15.791	1.006	15.681	0.853	0.397	0.693	0.109
Perímeter 2	17.066	1.442	17.004	1.328	0.152	0.880	0.062
Perímeter 3	19.725	1.868	19.763	1.887	−0.070	0.945	0.038
Perímeter 5	24.591	1.827	24.572	1.987	0.034	0.973	0.189
Perímeter 6	25.775	2.128	25.427	2.289	0.534	0.576	0.347
Perímeter 7	26.612	2.626	26.500	2.893	0.137	0.892	0.112
Perímeter 8	28.375	3.282	28.313	3.567	0.061	0.952	0.613
Perímeter 9	30.275	3.731	30.118	3.977	0.138	0.891	0.156
Perímeter 10	32.412	4.117	32.131	3.911	0.237	0.814	0.280
Perímeter 11	33.916	4.025	34.418	4.734	−0.274	0.786	0.501

Mean (*M*), Standard Deviation (*SD*).

**Table 4:** Mann-Whitney U. Perimeter measurement differences.

	Affected Hemibody		Healthy Hemibody		Mann–Whitney U
	*N*	Mid-Range	*N*	Mid-Range	*p*
Perimeter 4	24	22.96	22	24.09	0.775
Perímeter 12	2	2.50	2	2.50	0.999

**Table 5:** Mann-Whitney U. Pretest and post-test intergroup differences.

	Affected Hemibody		Healthy Hemibody		Mann–Wtihney U
	*N*	Mid-Range	*N*	Mid-Range	*p*
1RM unilateral pretest	24	22.94	22	24.11	0.763
Pre-test manual grip strength	24	22.44	22	24.66	0.571
Arm volume pre-test	24	24.25	22	22.68	0.692
Pre-test upper limb volume	24	24.33	22	22.59	0.660
1RM unilateral post	24	22.08	22	25.05	0.451
Post-test unilateral grip strength	24	22.65	22	22.33	0.934
Arm volume unilateral post-test	24	24.83	22	22.05	0.482
Unilateral upper limb volume post-test	24	24.46	22	22.45	0.613

**Table 6:** Mann–Whitney U. Intergroup differences.

	Not Lymphedema		Lymphedema		Mann-Whitney U
	*N*	Mid-Range	*N*	Mid-Range	*p*
Fat mass percentage	11	11.27	11	11.73	0.898
Water percentage	11	11.36	11	11.64	0.849

**Table 7:** T-test independent samples. Intergroup differences.

	Not Lymphedema		Lymphedema		T Student Independent Test	
	*N*	Mid-Range	*N*	Mid-Range	*p*	Effect Size
BMI	11	28.263	11	27.027	0.450	1.236
Weight	11	73.400	11	68.245	0.340	5.154
Fat mass kg	11	28.691	11	25.682	0.459	3.009
Muscle mass	11	42.436	11	40.391	0.227	2.045
Lean mass	11	44.709	11	42.564	0.227	2.145
Chest fly press	11	25.978	11	26.391	0.854	0.412
Bilateral contraction chest	11	26.391	11	23.504	0.424	2.886

**Table 8:** T-test-related samples. Intragroup comparison.

	Pre-Test		Post-Test		Student-Related Samples Test	
	*M*	*SD*	*M*	*SD*	*p*	Effect Size
Affected pectoral strength	25.514	8.060	27.310	7.813	0.009	8.371
Healthy forearm volume	663.198	110.935	691.917	97.116	0.092	68.241
Affected forearm volume	679.59	106.44	692.953	104.357	0.347	76.377
Muscle mass	41.617	3.932	40.217	4.316	0.002	1.868
Lean mass	43.852	4.129	42.378	4.543	0.002	1.966
Weight	71.143	12.170	71.178	12.159	0.861	0.938

Mean (*M*), Standard Deviation (*SD*).

**Table 9:** Wilcoxon test. Intragroup comparison.

	Wilcoxon
	*p*
Healthy pectoral strength	0.003
Grip affect side	0.047
Grip healthy side	0.032
Affected arm volume	0.092
Healthy arm volume	0.115
Affected upper limb volume	0.209
Healthy upper limb volume	0.077
Water percentage	0.291
Fat mass percentage	0.267
Fat mass kg	0.322

## Data Availability

Data for this research are available upon request from the corresponding author.
